# Subacromial Impingement Syndrome Caused by a Voluminous Subdeltoid Lipoma

**DOI:** 10.1155/2014/760219

**Published:** 2014-02-23

**Authors:** Jean-Christophe Murray, Stéphane Pelet

**Affiliations:** Division of Orthopaedic Surgery, CHU de Québec—Enfant-Jésus Hospital, 1401 18th Street, Québec, QC, Canada G1J 1Z4

## Abstract

Subacromial impingement syndrome is a clinical diagnosis encompassing a spectrum of possible etiologies, including subacromial bursitis, rotator cuff tendinopathy, and partial- to full-thickness rotator cuff tears. This report presents an unusual case of subdeltoid lipoma causing extrinsic compression and subacromial impingement syndrome. The patient, a 60-year-old man, presented to our institution with a few years' history of nontraumatic, posteriorly localized throbbing pain in his right shoulder. Despite a well-followed 6-months physiotherapy program, the patient was still suffering from his right shoulder. The MRI scan revealed a well-circumscribed 6 cm × 2 cm × 5 cm homogenous lesion compatible with a subdeltoid intermuscular lipoma. The mass was excised en bloc, and subsequent histopathologic examination confirmed a benign lipoma. At 6-months follow-up, the patient was asymptomatic with a complete return to his activities. Based on this case and a review of the literature, a subacromial lipoma has to be included in the differential diagnosis of a subacromial impingement syndrome refractory to nonoperative treatment. Complementary imaging modalities are required only after a failed conservative management to assess the exact etiology and successfully direct the surgical treatment.

## 1. Introduction

Subacromial impingement syndrome is a clinical diagnosis encompassing a spectrum of possible etiologies, including subacromial bursitis, rotator cuff tendinopathy, and partial- to full-thickness rotator cuff tears [1]. Despite some controversy regarding the significance of this entity [2], most authors recognize that subacromial impingement syndrome results from either intrinsic tendon degeneration or extrinsic compression, which can be primary or secondary. Primary structures responsible for extrinsic compression include the anterior acromion, coracoacromial ligament, and acromioclavicular joint. Secondary causes of impingement are multiple and can include tuberosity fracture nonunion or malunion, a mobile os acromiale, calcific tendinitis, instability, or iatrogenic factors [1]. This report presents an unusual case of subdeltoid lipoma causing secondary extrinsic compression and subacromial impingement syndrome.

## 2. Case Report

A 60-year-old man (a maintenance employee) presented to our institution with a few years' history of nontraumatic, posteriorly localized throbbing pain in his right shoulder. He also reported a traction injury to the same shoulder a few months ago while he was pulling on a cord trying to start a lawn mower. Since this episode, he was suffering a new, sharp pain in the anterolateral region of his shoulder and was now unable to fully accomplish his work. The patient first presented to our unit about 6 months after this working injury and was diagnosed with a rotator cuff tendinopathy. He was discharged with a reference for physiotherapy, and a follow-up appointment was scheduled 3 months later.

At follow-up, the patient was still suffering from his right shoulder despite a well-followed physiotherapy program. He reported a light attenuation in his symptomatology, mainly regarding the anterolateral pain. However, his posteriorly localized, long standing pain was still bothering him. The examination findings were of mild acromioclavicular joint tenderness on palpation. He also had pain at 90 degrees of active shoulder abduction, although his active range of motion was complete in all planes. Neer and Hawkins impingement tests were both positive. The Jobe supraspinatus test was negative for weakness but elicited some pain, and examination of all other rotator cuff muscles was normal. A “Popeye sign” indicative of a long head of biceps tendon rupture was also present on his arm. The neurovascular status of his upper extremity was unremarkable.

**Figure 1:**
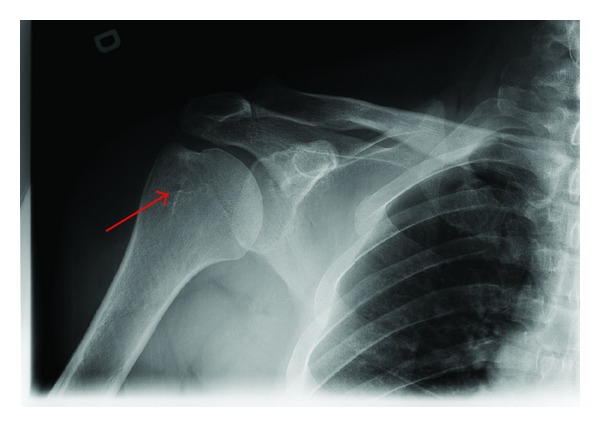
Plain radiograph of right shoulder showing small degenerative cysts at the site of insertion of the supraspinatus tendon on the greater tuberosity (arrow), indicative of rotator cuff tendinopathy.

**Figure 2:**
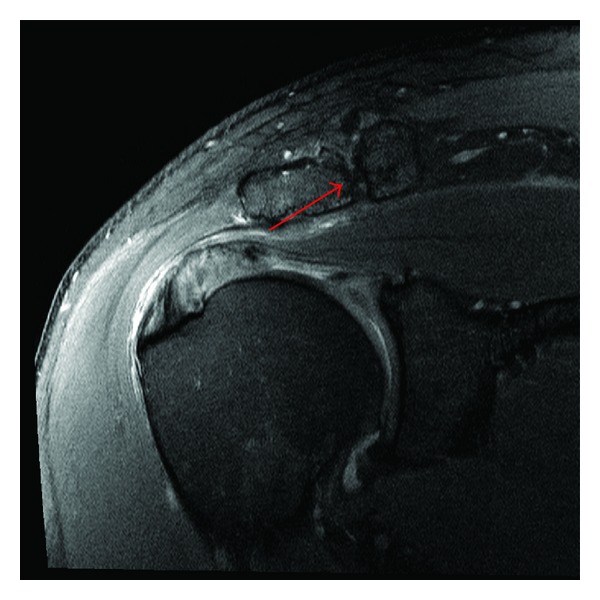
Coronal PD SPAIR magnetic resonance image of right shoulder showing moderate-to-severe osteoarthritis of the acromioclavicular joint (arrow), without any conflicting osteophytes in regard to the underlying rotator cuff.

**Figure 3:**
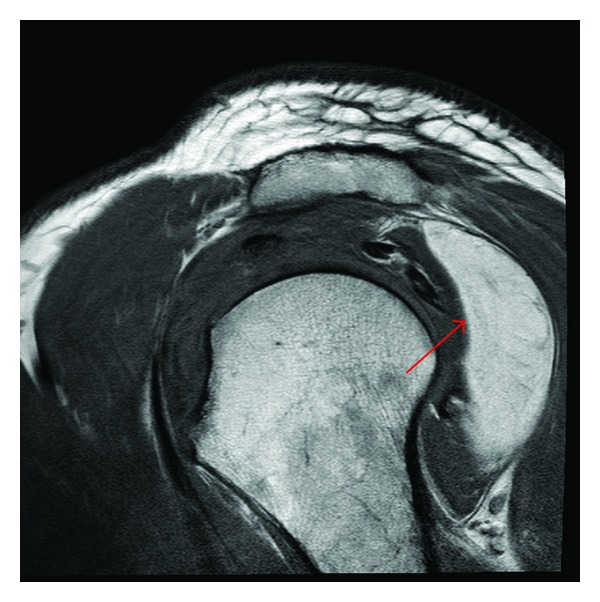
Sagittal T1 TSE magnetic resonance image of right shoulder showing a homogenous lesion (arrow) compatible with an intermuscular lipoma under the posterior aspect of the deltoid, extending to the undersurface of the acromion.

Plain radiographs were within normal limits except for some indirect signs of rotator cuff tendinopathy (Figure 1). An MRI scan revealed degenerative changes affecting the acromioclavicular joint indicative of a moderate-to-severe osteoarthritis but without significant osteophytes causing impingement on the underlying rotator cuff (Figure 2). There was also a rotator cuff tendinopathy of the subscapularis and a partial-thickness tear of the supraspinatus tendon, in addition to a long head of biceps tendon rupture. Most notably, the MRI scan revealed a well-circumscribed 6 cm × 2 cm × 5 cm homogenous mass with signal intensity resembling that of fat underlying the posterior deltoid. The mass was in close relation to the infraspinatus and the teres minor and was clearly extending under the acromion. There was no invasion of the tumor into the rotator cuff or into the surrounding tissues. The appearance of the lesion was compatible with a subdeltoid intermuscular lipoma without any radiologic sign of malignancy (Figure 3).

The patient was admitted for surgery. The operation was performed in the beach chair position under general anesthesia. An open distal clavicle resection and acromioplasty was first achieved through a superior incision centered over the acromioclavicular joint. The voluminous subdeltoid lipoma was then excised *en bloc* with a second incision cutting through the fibers of the posterior deltoid. The lesion was clearly invading the subacromial space at the time of the surgery. The histopathologic examination revealed a benign lipoma without any malignant features.

At 6-month postoperative follow-up, the patient was asymptomatic with normal shoulder function and a complete return to his activities.

## 3. Discussion

Subacromial impingement syndrome is a frequent cause of shoulder pain [3]. In his 1972 original report, Neer emitted the hypothesis that the anterior lip and undersurface of the anterior third of the acromion were the structures involved in the impingement of the rotator cuff [4]. The term *impingement syndrome* was then introduced to refer to the full spectrum of rotator cuff disease, before modern diagnostic modalities were widely available. The current availability of techniques such as sonography, magnetic resonance imaging, and arthroscopy now enables an accurate diagnosis to be made on an anatomic basis [2].

The clinical presentation of this case was compatible with a subacromial impingement syndrome, although the long-standing, posterior pain was somewhat atypical. No radiologic tests were available at the initial evaluation, and the diagnosis of rotator cuff tendinopathy was made solely on a clinical basis. The traumatic traction injury reported a few months ago resulted in an acute long head of biceps tendon rupture, which exacerbated the symptoms of the patient. Following a 3-month trial of conservative management, two factors were present as a possible etiology for the subacromial impingement syndrome. The acromioclavicular joint osteoarthritis was no longer retained as an etiology in the face of the absence of significant inferior osteophytes causing impingement on the underlying rotator cuff. The disease was nevertheless addressed at the time of surgery, since physical examination and radiologic tests were also consistent with symptomatic acromioclavicular osteoarthritis. Therefore, the voluminous subdeltoid lipoma was the only remaining etiology for the impingement syndrome, and this was in agreement with the MRI and peroperative findings of subacromial space invasion by the tumor.

We found only a few cases of impingement syndrome caused by a lipoma invading the subacromial space. Ferrari et al. [5] reported on a 45-year-old man suffering from an intramuscular supraspinatus lipoma causing impingement syndrome. Similarly, a lipoma of the supraspinatus muscle was also found as the cause of a subacromial impingement on a 51-year-old man [6]. Relwani et al. [7] reported on a 52-year-old woman with a subacromial lipoma impinging the underlying cuff and the biceps tendon. Most notably, these previous cases, and our patient as well, all have in common a failure of a well-followed conservative management before any additional investigations were performed. In all these cases, the key to the successful diagnosis of an impinging lipoma was a magnetic resonance imaging scan asked in this context. To our knowledge, our case is the only report of a posterior subdeltoid lipoma causing a clear picture of subacromial impingement syndrome.

Identifying the etiology of shoulder pain in subacromial impingement syndrome can be quite challenging, as the cause is usually multifactorial. Both intrinsic tendon degeneration and extrinsic compression may play a role. Most authors believe that rotator cuff disease usually arises from primary external compression by the anterior acromion, coracoacromial ligament, and acromioclavicular joint [1]. Secondary causes of external compression are even more difficult to diagnosis due to their rarity. In this case, the only finding on history suggestive of an unusual cause of impingement syndrome was the posteriorly localized pain. Magnetic resonance imaging provided the key information necessary to pinpoint the subdeltoid lipoma. This pathology should be included in the differential diagnosis of subacromial impingement syndrome with atypical characteristics or unresponsive to conservative management.

The diagnosis of subacromial impingement is often stated on a clinical basis of shoulder pain. The primary investigation should include plain radiographs of the involved shoulder. A proper nonsurgical management (analgesia, rest, physiotherapy, and modifying habits) is required for at least 6 months, as the cause is often an uncomplicated supraspinatus tendinopathy. In front of a failed conservative trial, the clinician should be alert to the possibility of an unusual entity causing secondary extrinsic compression with subacromial impingement syndrome, and this should warrant supplementary diagnosis imaging such as echography or magnetic resonance imaging. The treatment will then focus on the exact etiology of the subacromial impingement syndrome. An improper diagnosis can lead to an unsuccessful surgical treatment, for example, acromioplasty, which is commonly performed in front of a persistent impingement syndrome of unknown etiology.

## 4. Conclusion

Subacromial impingement syndrome is a frequent clinical diagnosis. Extrinsic etiologies are described, mainly related to the acromioclavicular joint. Based on this case and a review of the literature, a subacromial lipoma has to be included in the differential diagnosis. Complementary imaging modalities are required only after a failed conservative management to assess the exact etiology and successfully direct the surgical treatment. Liberal shoulder acromioplasty should not be performed without an accurate diagnosis as extrinsic etiologies are frequently overlooked.
